# Glutamatergic markers, age, intellectual functioning and psychosis in 22q11 deletion syndrome

**DOI:** 10.1007/s00213-015-3979-x

**Published:** 2015-06-10

**Authors:** Laurens J. M. Evers, Therese A. M. J. van Amelsvoort, Jaap A. Bakker, Mariken de Koning, Marjan Drukker, Leopold M. G. Curfs

**Affiliations:** Koraalgroup, MFCG, Panheelderweg 3, 6097 AH Heel, The Netherlands; Governor Kremers Centre, Maastricht University Medical Centre, Maastricht, The Netherlands; Department of Psychiatry and Psychology, School for Mental Health and Neuroscience MHeNS, Maastricht University Medical Centre, Maastricht, The Netherlands; Mondriaan Mental Healthcare, Heerlen, The Netherlands; Virenze Mental Healthcare, Gronsveld, The Netherlands; Department Clinical Genetics, Maastricht University Medical Centre, Maastricht, The Netherlands; Department of Clinical Chemistry and Laboratory Medicine, Leiden University Medical Centre, Leiden, The Netherlands; Department of Psychiatry, Academic Medical Centre, University of Amsterdam, Amsterdam, The Netherlands; Arkin Mental Health Care, Amsterdam, The Netherlands; CAPHRI, School for Public Health and Primary Care, Maastricht University, Maastricht, The Netherlands; GROW School for Oncology and Developmental Biology, Maastricht University, Maastricht, The Netherlands

**Keywords:** Proline, Glutamate, Glutamine, 22q11 deletion syndrome, Age, Cognition, Psychosis

## Abstract

**Rationale:**

Patients with 22q11 deletion syndrome (22q11DS) have a high prevalence of intellectual disabilities and psychiatric disorders, including psychosis. Haplo-insufficiency of genes in the deleted region may offer a partial explanation for the increased vulnerability for psychosis and intellectual disability. One gene of particular interest is the gene coding for proline dehydrogenase (PRODH), an enzyme responsible for the conversion of proline into glutamate.

**Objectives:**

Because abnormalities in glutamatergic signaling are thought to be responsible for cognition and psychosis in the general population, we hypothesized that PRODH haplo-insufficiency may underlie some of the cognitive and psychotic features seen in 22q11DS.

**Methods:**

In this explorative study, we investigated the relation between plasma proline, glutamate, and glutamine and age, intelligence, and psychosis in 64 adults with 22q11DS.

**Results:**

Hyperprolinemia was found in 31.3 % of subjects with 22q11DS. A relation between glutamine, glutamate, proline, and presence of psychosis was not observed. Regression analysis revealed a positive relation between plasma glutamate and age, a positive relation of glutamate with antipsychotic drugs, a relation of glutamine and gender, and a positive relation of glutamine and mood stabilizing drugs, and a negative relation of the ratio glutamine/glutamate and age. The group with relatively lower IQ had higher glutamate levels compared to the group with relatively higher IQ.

**Conclusions:**

Our results suggest that 22q11DS is accompanied by abnormalities in glutamatergic metabolism. Future longitudinal studies are needed to further investigate the glutamatergic system in 22q11DS and how this affects the development of cognitive problems and psychopathology.

## Introduction

22q11 deletion syndrome (22q11DS) is a genetic disorder caused by a microdeletion on the long arm of chromosome 22. Its prevalence is estimated to be 1:4000 (McDonald McGinn and Sullivan 2011). Besides a variety of physical symptoms, patients with 22q11DS frequently suffer from psychiatric disorders (Hiroi et al. 2013; Schneider et al. 2014). There has been a particular interest in schizophrenia because patients with 22q11DS have a 25–30 times higher risk of developing schizophrenia (Murphy et al. 1999). In addition, an increased prevalence of a 22q11 deletion (0–5.3 %) among patients with schizophrenia has been observed in some studies (Sporn et al. 2004; Wiehahn et al. 2004) but not in others (Hoogendoorn et al. 2008). Especially, the high prevalence of schizophrenia in people with 22q11DS has led to speculations about how a reduced dosage of genes located at 22q11 could be implicated in the etiology of schizophrenia. One gene of particular interest is the gene coding for proline dehydrogenase (PRODH), an enzyme responsible for the conversion of proline (Pro) into glutamate (Glu). Because of reduced PRODH gene dosage, it is expected that people with 22q11DS have reduced PRODH enzyme activity and increased Pro levels (Goodman et al. 2000; Magnee et al. 2011; Raux et al. 2007). Increased Pro levels (plasma Pro 350–550 μmol/L) may be a risk factor for schizo-affective disorders (Jacquet et al. 2005). Type 1 hyperprolinemia resulting from an inherited PRODH deficiency is a disease characterized by severe hyperprolinemia (>550 μmol/L), seizures, intellectual disability, and psychiatric symptoms (Jacquet et al. 2002).

Taken together, it seems plausible that high Pro levels could play a role in the development of psychosis, neurological, and cognitive deficits in 22q11DS. Pro, a precursor of Glu, has been the subject of several studies in 22q11DS (Goodman et al. 2000; Raux et al. 2007; Vorstman et al. 2009), and all conclude that hyperprolinemia is common in 22q11DS patients.

Glu is the principal excitatory neurotransmitter in the brain (Nedergaard et al. 2002) and plays an important role in learning, memory (Cotman et al. 1988), emotional regulation, and motivational behavior (Mora and Cobo 1990). However, excessive concentrations of Glu are toxic and result in cell death (Lau and Tymianski 2010). Abnormal Glu neurotransmission is thought to play a crucial role in epilepsy and several chronic neurodegenerative disorders, including Parkinson’s disease (Marsman et al. 2013; Mehta et al. 2013). Both Parkinson’s disease (Booij et al. 2010; Butcher et al. 2013) and epilepsy (Mori et al. 2011) have been associated with 22q11DS. Moreover, pharmacological treatment for these disorders sometimes targets the glutamatergic system (Bleich et al. 2003). The role of the glutamatergic neurotransmission in the etiology of schizophrenia has received increased interest, in addition to the existing dopaminergic theory. Molecular imaging studies in schizophrenia point to an excess of dopamine in the associative striatum, explaining mainly positive symptoms, and a hypodopaminergic state in the prefrontal cortex (Stone et al. 2007). However, Kambeitz et al. (2014) recently stated that there is insufficient in vivo evidence of altered dopaminergic function in cortical and extrastriatal regions that could explain negative or cognitive symptoms. It is now hypothesized that decreased glutamatergic neurotransmission via hypofunctional NMDA receptors leads to increased prefrontal glutamatergic activity in schizophrenia, possibly contributing to negative cognitive symptoms (Poels et al. 2014). A recent meta-analysis (Song et al. 2014) shows that the peripheral measured Glu in patients with schizophrenia is elevated compared to controls. Compared to peripheral levels, levels in the brain, measured with magnetic resonance spectroscopy (MRS), vary with different brain regions (Marsman et al. 2013). Peripheral levels of Glu are therefore not necessarily related to levels measured in the brain. High peripheral levels of glutamate have been considered a trait marker for schizophrenia (De Luca et al. 2008).

There is limited in vivo research on the function and modulation of the glutamatergic system in adult 22q11DS patients. The only MRS study in adult 22q11DS patients with schizophrenia showed that, compared to 22q11DS patients without schizophrenia, Glu was increased in the hippocampus, with no differences in Glu concentrations in the dorsolateral prefrontal region (da Silva Alves et al. 2011). To our knowledge, plasma glutamine (Gln) levels have only been investigated in 22q11DS in a small sample group (*n* = 9) by da Silva Alves et al. (2011). This study did not find any differences in Gln or Pro plasma levels between 22q11DS subjects with and without psychosis. Because of its role, both in psychosis and in cognition in this study, we assessed Glu, Gln, the ratio of Gln/Glu, and Pro in plasma of adult 22q11DS patients and hypothesized that (a) Pro, Glu, and Gln levels are related to the presence of psychosis; (b) Pro, Glu, and Gln levels are related to intellectual functioning; (c) hyperprolinemia is highly prevalent among adult 22q11DS patients.

## Methods

The study was approved by the Medical Ethics Committee of the University of Maastricht, Maastricht, and the University of Amsterdam, Amsterdam, the Netherlands, and is part of an ongoing multi-center research project on neurotransmitter functioning in 22q11DS adults.

## Subjects

Participants were recruited through the Dutch 22q11DS family network, a specialized psychiatric 22q11DS outpatient clinic, and through several intellectual disability centers in the Netherlands. A subset of this group enrolled previously in an earlier study by Raux et al. (2007), in which Pro was the only investigated amino acid. Because we have extended the original cohort of adults and added Glu, Gln, and the ratio of Gln/Glu as outcome measures, we were able to address the complex interplay of cognitive level and psychosis that are present in 22q11DS. Sixty-four patients with a confirmed deletion at chromosome 22q11.2 were included. Exclusion criteria were age below 18 years and medical conditions that affect brain functions not associated with 22q11DS, e.g., Alzheimer’s disease. All patients and their carers were informed about the study, and written informed consent was obtained.

## Clinical assessment

### IQ measurements

Full-scale IQ (FSIQ) scores were obtained in the 22q11DS group using a shortened version of the Wechsler Adult Intelligence Scale (WAIS) version III (Wechsler 1997). Patients unable to perform the WAIS because of their low intelligence were investigated using a Vineland screener (Scholte et al. 2008). The outcome of this test was converted to a FSIQ rating as described earlier (Evers et al. 2014b; Kraijer and Plas 2006).

### Psychopathology outcome measures

All patients were assessed for lifetime presence of a psychotic disorder based on information obtained from medical records and/or the present score on the Mini Psychiatric Assessment Schedules for Adults with Developmental Disabilities (PAS-ADD) (Prosser et al. 1998) or the Mini-International Neuropsychiatric Interview (MINI) (Sheehan et al. 1998). The mini PAS-ADD was used in patients functioning at a level below IQ 55, and the remaining were evaluated with the MINI.

### Use of psychotropic drugs

The dosage of prescribed antipsychotic drugs was rated according to haloperidol equivalents. The use of mood stabilizing drugs was noted as present or absent.

## Amino acid analysis

Blood samples were obtained by venipuncture for the determination of amino acids (Pro, Gln, and Glu). The samples were cooled on ice and centrifuged, and plasma was frozen at −20 °C until analysis. Concentrations in plasma were determined using a standardized procedure for the quantification of amino acids in biological fluids. Analyses were performed using ultra performance liquid chromatography combined with tandem mass spectrometry (Acquity UPLC-Micromass Quattro Premier XE TandemMass Spectrometer (Waters, Milford, MA)) (Waterval et al. 2009). Local reference ranges were used in line with published values (Blau et al. 2008). The ratio Gln/Glu was defined as the concentration of Gln divided by Glu concentration.

## Statistical analysis

All statistical analyses were performed using Stata version 12.1 (StataCorp 2011). Mean levels of amino acids with distribution and reference range were obtained. Descriptive statistics were used to determine percentages of high Pro levels (hyperprolinemia) as described by Jacquet et al. (2005). The relation of psychosis with Pro, Glu, Gln, and the ratio Gln/Glu was investigated with a regression analysis which was performed in four separate models with Glu, Gln, the ratio of Gln/Glu, and Pro, respectively, as dependent variables. Age, use of antipsychotic medication, presence of lifetime psychosis, gender, and use of mood stabilizing drugs were the main independent variables in all models. We have not corrected for multiple comparison due to a limited power. Because of the use of two different methods to establish intelligence (Vineland-S in the lower functioning group in contrast to the WAIS in the relatively higher functioning group (FSIQ > 55)), the method refers automatically to subgroups of higher and lower intelligence and is shown to act as a powerful confounder or as a mediator. We, therefore, analyzed only the dichotomized IQ. If IQ was assessed with the Vineland screener rather than the WAIS version III, subjects were categorized in the “lower IQ” category. To establish the relation of Glu, Gln, and the ratio Gln/Glu with intelligence, we performed a *t* test analysis to compare amino acid levels in “lower IQ” and “higher IQ” subgroups.

## Results

Thirty (47 %) of the patients were male. Mean age was 33.7 years (SE 1.1, range 18–59) (Table 1). Twenty-nine out of 64 patients (45 %) had a history of psychosis, and 29 out of 64 (45 %) used prescribed antipsychotic drugs. Eight out of the 64 patients (13 %) had both a psychotic disorder and a depressive disorder diagnosed; twenty-six out of the 64 patients (40 %) were diagnosed with only a psychotic disorder and 3 out of 64 patients (5 %) only with a depressive disorder. Twenty-seven out of 64 patients had no psychiatric diagnosis (42 %). Presence of depressive disorder (alone or combined with psychosis) was not related to Pro, Glu, or Gln. Twenty out of 64 patients (31.3 %) had hyperprolinemia (proline 316–550 μmol/L in female and 377–550 in male), and six of these had severe hyperprolinemia, (proline >550 μmol/L) (9.4 %) (Jacquet et al. 2005). In 13 out of the 64 subjects, glutamatergic values were not available because they were a subset from the original Raux et al. study (2007) and did not include glutamatergic metabolites. One out of 53 subjects had a Glu level above the reference range, and another one out of 53 subjects had a Gln level below the reference range.

**Table 1:** Descriptives

	N	Mean	SE	Range	
FSIQ	64	51.8	3.19	7–96	
Age (years)	64	33.7	1.1	18–59	
Sex: M:F	64	30:34			
Treated with antipsychotic drugs	29/64 (45 %)				
Treated with mood stabilizing drugs	20/64 (31 %)				
History of psychosis	29/64 (45 %)				
Amino acids	N	Level (mean)	SE	CI	reference range
Proline (μmol/L)^a^	64	316.3	18.6	279.1–353.6	77–316 (♀) 77–377 (♂)
Glutamate (μmol/L)^b^	51	53.33	3.2	48.9–61.7	<121
Glutamine (μmol/L)^c^	51	529.9	12.7	504.5–555.3	344–743
Ratio glutamine/glutamate	51	11.2	0.79	9.6–12.8	

^a^Twenty out of 64 subjects (31.3 %) had hyperprolinemia (Pro 350–550 μmol/L), six of them severe hyperprolinemia (Pro >550 μmol/L) (9.4 %) (Jacquet et al. 2005)

^b^One out of 51 had glutamate above normal level

^c^One out of 51 had glutamine below normal level

In the regression models, psychosis was not associated with any of the four outcome variables (Table 2). Age was negatively associated with Gln and ratio of Gln/Glu and positively associated with Glu. Glu was positively related with dosage of antipsychotic medication (Table 2). Gender affected Glu and Gln with higher Glu and Gln in men compared to women (50.9 and 569 vs. 50.6 and 488). Pro, Glu, Gln, or the ratio Gln/Glu were not significantly related to any other included variables.

**Table 2:** Regression analysis in four models with age, antipsychotic drugs life time psychosis and use of mood stabilizing drugs as dependent variables

		Coef	S.E.	*p* value	95 % CI
Proline	Age	−1.02	2.12	0.63	−5.28–3.24
	Antipsychotic drugs^b^	5.15	7.03	0.47	−8.96–19.26
	Life time psychosis	−81.55	46.93	0.09	−175.81–12.72
	Gender	−78.81	42.92	0.07	−165.02–7.39
	Mood stabilizing drugs	−56.78	52.74	0.29	−162.70–49.14
Glutamate	Age	0.80	0.32	.018*	0.14–1.46
	Antipsychotic drugs^b^	2.07	1.01	.046*	0.04–4.10
	Life time psychosis	−6.68	6.94	0.34	−20.71–7.35
	Gender	−11.18	6.64	0.10	−24.95–−2.25
	Mood stabilizing drugs	3.29	8.03	0.68	−12.95–19.52
Glutamine	Age	−1.18	1.10	0.29	−3.40–1.04
	Antipsychotic drugs^b^	3.06	3.41	0.38	−3.84–9.95
	Life time psychosis	14.17	23.50	0.55	−33.37–61.72
	Gender	−65.17	22.50	0.006*	−110.53–−19.51
	Mood stabilizing drugs	68.17	27.19	0.016*	13.17–123.18
Ratio Gln/Glu	Age	−0.14	0.06	0.003*	−0.27–−0.02
	Antipsychotic drugs^b^	−0.19	0.19	0.34	−0.58–0.21
	Life time psychosis	1.07	1.35	0.43	−1.65–3.79
	Gender	1.43	1.29	0.28	−1.18–4.03
	Mood stabilizing drugs	2.06	1.56	0.19	−1.09–5.12

*p ≤ 0.05

^b^In haloperidol equivalents

In the subgroup analysis, *t* tests revealed that Glu was higher and ratio Gln/Glu lower in the Vineland lower IQ group compared to the WAIS higher IQ group (Table 3). There were no between-group differences in Pro levels and Gln levels.

**Table 3:** *t* test analyses of proline, glutamate, glutamine, and ratio glutamine/glutamate between different IQ measurement groups

Mean (SE)	WAIS group	Vineland-group	*t*	*p* value
Proline (μmol/L)	338.8 (31.6)	302.3 (22.8)	0.95	0.345
Glutamate (μmol/L)	46.1 (3.3)	62.3 (4.4)	−2.57	.0133*
Glutamine (μmol/L)	551.1 (19.6)	517.3 (16.3)	1.30	0.2007
Ratio glutamine/glutamate	13.0 (1.2)	9.4 (0.72)	2.70	.0097*

*p < 0.05

## Discussion

This explorative study is the first describing plasma levels of Glu, Gln, Pro, and ratio Gln/Glu in a group of adult 22q11DS patients.

While in schizophrenia, high ratios of Gln/Glu have been found in different brain regions (Shirayama et al. 2010), as well as high peripheral levels of Glu (Song et al. 2014; Tomiya et al. 2007); we did not observe a relation between Gln/Glu, Gln, Glu and Pro, and presence of psychosis. These results reject our hypotheses concerning psychosis and its relation with Glu, Gln, and Pro in our study. We showed, in a *t* test analysis, between-group differences in Glu and ratio Gln/Glu with higher Glu and a lower ratio Gln/Glu in the lower IQ group. The relation of higher Glu and lower Gln/Glu ratios in the lower IQ group supports the hypothesis that Glu and Gln levels may be related to intellectual functioning. Because intellectual decline is a common feature in 22q11DS (Duijff et al. 2013; Evers et al. 2014b) and Glu has neurotoxic proportions, high Glu levels may play a role in this decline. As far as we know, the relationship between peripheral ratio Gln/Glu and IQ in schizophrenia has never been investigated. Though not hypothesized, we found that Glu levels were positively, and ratio of Gln/Glu negatively, associated with age. Our Glu findings are in contrast with the literature in the general population, which suggests an age-related decline in Glu or no age-related change (Marsman et al. 2013; Sailasuta et al. 2008). An MRS study in the general population showed that Gln tended to increase with age (Kaiser et al. 2005), whereas our findings in plasma were in the opposite direction. Most age-related findings are from MRS studies, so comparisons with our results have to be interpreted with caution. The gender difference we observed, i.e., higher Glu and Gln levels in men, is also in contrast with the finding of Hädel et al. (2013). They demonstrated in a MRS study in the general population that females had higher Glu levels in the hippocampal region compared to males, whereas in the cortical region, no differences were found. Jacquet et al. (2005) found higher Pro levels in plasma in healthy male subjects. In our study, we were unable to demonstrate a gender difference in Pro levels (*p* = 0.07), which may have been caused by the smaller sample size of our group.

We found a positive relation between Glu levels and dosage of antipsychotic drugs and a positive relation between Gln levels and the use of mood stabilizing drugs. The positive relation of Glu with antipsychotic medication is in contrast to findings in MRS studies in the normal population where antipsychotic medication seemed to correct increased Glu levels in patients with schizophrenia (Poels et al. 2014). In a 22q11DS MRS study (da Silva Alves et al. 2011), a positive correlation was found between dosage of drugs and Gln concentrations in the dorsolateral prefrontal cortex, not in the hippocampus. Antipsychotic drugs are likely to correct increased Glu levels in schizophrenia (Goff et al. 2002). In 22q11DS psychosis, it is sometimes difficult to treat (Kiehl et al. 2009), which theoretically could be indicative of antipsychotic drugs failing to correct Glu excess in 22q11DS. Butcher et al. (2015), however, demonstrated recently that patients with schizophrenia (half of them with 22q11DS) responded equally on clozapine treatment. Clozapine is the preferable antipsychotic treatment for treatment-resistant schizophrenia (Warnez and Alessi Severini 2014) and also in 22q11DS (Butcher et al. 2015). Clozapine has a receptor profile distinct from most antipsychotic drugs. In a recent study, patients with schizophrenia were compared depending on how they reacted to treatment with clozapine (Goldstein et al. 2015), indicating differences in glutamate levels between subgroups. We further hypothesized that hyperprolinemia is highly prevalent among adult 22q11DS patients. We confirmed that hyperprolinemia is a common finding in adults with 22q11DS. Twenty out of 64 subjects (31.3 %) were hyperprolinemic, with six of them severely hyperprolinemic (9.4 %). This is consistent with findings in two earlier studies in children and adults (50 and 37 % hyperprolinemia) (Goodman et al. 2000; Raux et al. 2007) and is likely to be the result of hemizygosity of the PRODH gene in 22q11DS. We failed to confirm the hypothesis that high Pro levels may be related to psychosis and to intellectual functioning in the total 22q11DS group or in the subgroups. One previous study found a negative relation between Pro and intelligence scores (Raux et al. 2007), a finding we could not replicate and we are unable to explain. One possibility is that, compared to Raux et al. (2007), we included more 22q11DS patients with a low IQ and our sample had a lower mean IQ (mean IQ of 64 vs. 53).

Based on our findings regarding glutamatergic alterations in 22q11DS and our earlier observations on dopamine and serotonin (Evers et al. 2014a), it would be interesting to consider new pharmacological strategies. These may include drugs targeting the glutamatergic system, combined with dopaminergic, noradrenergic, and serotonergic action.

### Strength and limitations

This study is the first investigating plasma amino acids in 22q11DS including Pro, Glu, Gln, and the ratio Gln/Glu. However, it has some limitations. With the inclusion of 22q11DS patients that function at a lower intellectual level, we cover a broader range of this syndrome; however, it cannot be ruled out that low FSIQ is overrepresented. The use of two different methods to establish intelligence was unavoidable (IQs below 55 cannot be established by Wechsler instruments) but is a serious limitation in this study. The amino acids were measured in plasma, which is an indirect method, and caution in comparison with studies measuring directly in cerebrospinal fluid (CSF) or with MRS is recommended. However, peripheral concentrations of Glu seem to be positively correlated to Glu CSF levels (Alfredsson et al. 1988). It would be more informative to measure directly in the brain with CSF measurements or MRS techniques, but these techniques are more invasive and are difficult to perform in 22q11DS patients with a low IQ. Therefore, comparison of our results with results in MRS studies or in CSF studies should be interpreted with caution. Another limitation of this study is the lack of a control group and the relatively small sample size in the subgroups.

### Future/concluding remark

The reported glutamatergic levels show an opposite pattern compared to those reported in the general population, and this may explain the cognitive problems in 22q11DS. Longitudinal studies in patients with 22q11DS are needed to follow the course of intellectual function and psychosis with special interest for the role of neurotransmitter systems. More insight into these mechanisms may result in possibilities for early treatment to prevent severe complications in this syndrome. Also, it will give us more insight into mechanisms of psychosis and intellectual decline in the normal population.
